# *Aliarcobacter butzleri* from Water Poultry: Insights into Antimicrobial Resistance, Virulence and Heavy Metal Resistance

**DOI:** 10.3390/genes11091104

**Published:** 2020-09-21

**Authors:** Eva Müller, Mostafa Y. Abdel-Glil, Helmut Hotzel, Ingrid Hänel, Herbert Tomaso

**Affiliations:** Institute of Bacterial Infections and Zoonoses (IBIZ), Friedrich-Loeffler-Institut, Federal Research Institute for Animal Health, 07743 Jena, Germany; Eva.Mueller@fli.de (E.M.); Helmut.Hotzel@fli.de (H.H.); hedwighaenel@web.de (I.H.); Herbert.Tomaso@fli.de (H.T.)

**Keywords:** *Aliarcobacter*, emerging pathogen, antibiotic susceptibility, whole-genome sequencing, antimicrobial resistance, virulence, heavy metal

## Abstract

*Aliarcobacter butzleri* is the most prevalent *Aliarcobacter* species and has been isolated from a wide variety of sources. This species is an emerging foodborne and zoonotic pathogen because the bacteria can be transmitted by contaminated food or water and can cause acute enteritis in humans. Currently, there is no database to identify antimicrobial/heavy metal resistance and virulence-associated genes specific for *A. butzleri*. The aim of this study was to investigate the antimicrobial susceptibility and resistance profile of two *A. butzleri* isolates from Muscovy ducks (*Cairina moschata*) reared on a water poultry farm in Thuringia, Germany, and to create a database to fill this capability gap. The taxonomic classification revealed that the isolates belong to the *Aliarcobacter* gen. nov. as *A. butzleri* comb. nov. The antibiotic susceptibility was determined using the gradient strip method. While one of the isolates was resistant to five antibiotics, the other isolate was resistant to only two antibiotics. The presence of antimicrobial/heavy metal resistance genes and virulence determinants was determined using two custom-made databases. The custom-made databases identified a large repertoire of potential resistance and virulence-associated genes. This study provides the first resistance and virulence determinants database for *A. butzleri*.

## 1. Introduction

The genus *Arcobacter* was included in the family *Campylobacteraceae* in 1991 [1]. The genus *Arcobacter* has recently been reorganized and is now separated into six different genera: *Arcobacter*, *Aliarcobacter*, *Halarcobacter*, *Malaciobacter*, *Poseidonibacter*, and *Pseudarcobacter* [2,3,4,5]. The twenty-nine validly published *Arcobacter* spp. are distributed over those genera, with *Arcobacter butzleri* (now *Aliarcobacter butzleri*) belonging to the genus *Aliarcobacter* (*A*.) [2,3,4,6,7].

*A. butzleri* usually causes self-limiting acute enteritis associated with watery diarrhea, nausea, abdominal pain and sometimes fever in humans [8,9]. Diarrhea occurs due to epithelial barrier dysfunction induced through changes in tight-junction proteins and the induction of epithelial apoptosis [10]. A long-term study showed that *A. butzleri* is the fourth most common *Campylobacter*-like organism isolated from human feces [11]. In rare cases, *A. butzleri* may cause bacteremia [8,12]. In animals, *A. butzleri* has been associated with enteritis and/or diarrhea in pigs, cattle, and horses, but has also been found in feces of healthy animals [9,13,14]. 

*A. butzleri* is the most prevalent *Aliarcobacter* species detected in food [15]. These bacteria have been isolated in products of animal origin such as poultry meat, pork and beef meat, but also in water, milk, dairy products, shellfish, and vegetables [9,16,17,18,19]. The consumption of contaminated food or water is the most probable route of transmission to humans and animals [8,9,20,21,22]. Contact with companion animals is also a possible way of transmission to humans as *A. butzleri* has been found in the oral cavity of cats [9,23]. Therefore, *A. butzleri* is not only an emerging foodborne and zoonotic pathogen [8,9], it is also considered as a serious hazard to human health [24]. However, in animals, horizontal and vertical transmission have been described [19,25].

Previous studies mostly examined the antibiotic susceptibility of *A. butzleri* phenotypically, but in the last few years the underlying antimicrobial resistance (AMR) genes have also been studied more closely [18,26,27,28,29,30]. Recently, more virulence-associated genes have been identified in *A. butzleri* in addition to the previously known homologous genes found in *Campylobacter jejuni* (*pldA*, *mviN*, *irgA*, *iroE*, *ciaB*, *hecA*, *hecB*, *cj1345*, *cadF*, *tlyA*) [18,26,27,31]. The resistance of *A. butzleri* to heavy metals has hardly been investigated to date [18,27]. 

Herein, we describe the antibiotic susceptibility profiles and the genomic characterization of two *A. butzleri* strains isolated from Muscovy ducks (*Cairina moschata*) from a water poultry farm in Thuringia, Germany. In addition, we describe the presence of putative AMR and heavy metal resistance genes as well as potential virulence determinants. Furthermore, we provide a database containing all currently known resistance and virulence-associated genes, which is easily accessible for all researchers to enable faster resistance profiling of *A. butzleri*.

## 2. Materials and Methods 

The strains 16CS0817-2 and 16CS0821-2 were isolated from two fecal samples obtained from a water poultry farm in Thuringia, Germany, in 2016. Each isolate originated from a single fecal sample of a Muscovy duck (*C. moschata*). The fecal samples were taken by a veterinarian with the permission of the animal owners. 

For this study, no ethical review process was required, as there were no experiments with animals as defined by the German Animal Protection Law (Tierschutzgesetz) and the Animal Welfare Laboratory Animal Regulation (Tierschutz-Versuchstierordnung).

### 2.1. Culturing and Identification

The *Aliarcobacter* isolates from fecal samples were cultivated in *Arcobacter* broth (Oxoid GmbH, Wesel, Germany) which was supplemented with three different antibiotics (cefoperazone, amphotericin, and teicoplanin (CAT), Oxoid GmbH). The broth was then spread on plates (Mueller–Hinton agar/CAT/5% defibrinated bovine blood, Sifin GmbH, Berlin, Germany). The incubation criteria for each step were: 48–72 h, 30 °C and microaerophilic atmosphere (5% O_2_, 10% CO_2_, and 85% N_2_). Suspicious colonies were further cultivated and then identified by matrix-assisted laser desorption/ionization time-of-flight mass spectrometry (MALDI-TOF MS) as described before [32,33]. IVD Bacterial Test Standard and Biotyper 3.1 software were used (Bruker Daltonik GmbH, Bremen, Germany). DNA was purified using the High Pure PCR Template Preparation Kit (Roche Diagnostics, Mannheim, Germany) following the manufacturer’s instructions, and the species identification was confirmed with a multiplex PCR assay [34].

### 2.2. Antimicrobial Susceptibility Testing

The antibiotic susceptibility was determined by using the gradient strip diffusion method (E-Test^TM^, bioMérieux, Nürtingen, Germany) following the manufacturer’s instructions. Briefly, the *Aliarcobacter* strains were transferred to Mueller–Hinton agar plates (Sifin GmbH) and incubated for 48–72 h at 30 °C under microaerophilic conditions. Then, the bacterial colonies were added to five milliliters of *Arcobacter* broth and incubated at 30 °C under microaerophilic conditions for a further 48–72 h. Subsequently, 750 µL were spread on Mueller–Hinton agar plates and one antibiotic gradient strip was placed on each plate. Each strain was tested against erythromycin (0.015–256 µg/mL, MA0108F, Oxoid GmbH), ciprofloxacin (0.002–32 µg/mL, MA0104F, Oxoid GmbH), streptomycin (0.064–1.024 µg/mL, REF: 526800, bioMérieux), gentamicin (0.06–1.024 µg/mL, MA0117F, Oxoid GmbH), tetracycline (0.015–256 µg/mL, MA0105F, Oxoid GmbH), doxycycline (0.016–256 µg/mL, REF: 142328, bioMérieux), ampicillin (0.016–256 µg/mL, REF: 412253, bioMérieux) and cefotaxime (0.002–32 µg/mL, REF:412281, bioMérieux). The minimum inhibitory concentration (MIC) was determined after 48 h of incubation at 30 °C under microaerophilic conditions. The strain *A. butzleri* DSM 8739 was used as a control.

In this study, the cut-off values for *Campylobacter* spp. provided by EUCAST [35] were used for erythromycin, ciprofloxacin, doxycycline, and tetracycline, as no specific breakpoints are available for *Aliarcobacter* spp. For gentamicin, ampicillin, and cefotaxime, we used the 2019 EUCAST breakpoints for *Enterobacterales*. For streptomycin, the cut-off values for *Campylobacter* spp. provided by the European Food Safety Authority were used [36]. The bacterial strains were classified as sensitive or resistant.

### 2.3. DNA Extraction and Whole-Genome Sequencing

Colony material from one to two plates was needed to obtain sufficient bacterial mass for DNA preparation. Each plate was washed with 2 mL of phosphate-buffered saline (PBS) and the liquid was collected in a 2-mL tube. The tubes were centrifuged for 20 min at 5400 rpm and the supernatant was discarded. The remaining content was washed at least twice with PBS buffer until the supernatant was clear. The resulting pellet was used for DNA recovery using the High Pure PCR Template Preparation Kit. The concentration of the double-stranded DNA (dsDNA) was examined with a Qubit 3 Fluorometer using the Qubit^TM^ dsDNA HS Assay Kit (both Invitrogen^TM^, ThermoFischer Scientific, Berlin, Germany). The Nextera XT DNA Library Preparation Kit (Illumina, Inc., San Diego, CA, USA) was used according to the manufacturer’s instructions to generate a sequencing library. Sequencing was done with an Illumina MiSeq instrument (Illumina, Inc., San Diego, CA, USA).

### 2.4. Bioinformatic Analyses

The raw data were assembled using SPAdes version 3.12.0 [37] after removing low-quality reads and sequencing primers with Trimmomatic (parameters are PE -phred33 LEADING:10 TRAILING:10 MINLEN:30 ILLUMINACLIP:adapters.fa:1:30:11) [38]. Only contigs larger than 500 base pairs (bp) and with a k-mer coverage > 5 were kept using an in-house script. The quality was then assessed with QUAST version 4.3 [39]. The Prokka annotation pipeline 1.14 was used in standard settings for annotation [40].

To confirm the species identity of the assembled data, taxonomic classification was performed using Kraken2 version 2.0.7 [41]. Furthermore, the average nucleotide identity (ANI) of the German strains was calculated in comparison to 11 *A. butzleri* genomes (including the *A. butzleri* reference genome RM4018), the reference genomes of *A. cryaerophilus* ATCC 43158^T^ and *A. trophiarum* LMG 25534^T^, as well as to the out-group genomes *Campylobacter* (*C.*) *jejuni* subsp. *jejuni* NCTC 11168^T^ and *Helicobacter* (*H.*) *pylori* NCTC 11637^T^, which were downloaded from the NCBI repository, using pyani version 0.2.9 [42]. In silico DNA–DNA hybridization (DDH) was done using the Genome-to-Genome Distance Calculator (GGDC) software [43]. In this study, the recommended formula 2 was used for analysis [43].

Multilocus sequence typing (MLST) based on the whole-genome sequences was done using the PubMLST database (pubmlst.org/arcobacter/) and the MLST tool version 2.15.2 with default settings [44].

In this study, public AMR databases were searched for known AMR genes and mutations, using ABRicate version 0.8.10, which uses the BLASTN algorithm. ABRicate includes, besides others, the databases ResFinder, CARD, ARG-ANNOT, and NCBI [45,46,47,48].

For the construction of the databases, previously described AMR, virulence-associated and heavy metal resistance genes were used [18,26,27]. Additionally, more resistance genes were added after screening the Prokka annotated assemblies of the genomes RM4018p, Ab_2211p, Ab_4511p, BMH_AB_233Bp, BMH_AB_246Bp, D4963p and L353p provided by Isidro et al. [26]. The genes derived from previous studies and the genes identified in this study were blasted with BLASTX using the non-redundant protein sequences (nr) database. Afterwards, all genes were extracted from those genomes with Geneious Prime^®^ 2019.2.3 [49], and were put together into two custom databases (ARCO_IBIZ_AMR; ARCO_IBIZ_VIRULENCE; both available at https://gitlab.com/FLI_Bioinfo_pub) within ABRicate. The here generated whole-genome sequences were screened within this database to identify the presence or absence of the genes. With a detection value of at least 50% coverage and 75% identity, a gene was considered to be present.

In addition, the *gyrA* gene, the 23S rRNA gene, the *rplV* gene and the *rplD* gene of the two strains were extracted using Geneious Prime^®^ 2019.2.3 to identify any known mutations.

The original contributions presented in this study are publicly available. The data has been deposited at DDBJ/ENA/GenBank under the accession WCIX00000000 and WCIY00000000. The version described in this study is version WCIX01000000 and WCIY01000000 (Bioproject: PRJNA575341).

## 3. Results and Discussion

### 3.1. Identification and Antimicrobial Susceptibility Testing

The two strains were identified by MALDI-TOF MS and multiplex PCR as *A. butzleri*. The species identification using MALDI-TOF MS (scores > 2.3) was reliable as there were sufficient spectra available for *A. butzleri* in the database for comparison, and the multiplex PCR assay was able to identify the species *A. butzleri* with 100% reliability [34,50].

As shown in Table 1, the tested *A. butzleri* isolates were susceptible to ciprofloxacin, ampicillin and gentamicin.

Most studies reported that *A. butzleri* is susceptible or shows low resistance rates to gentamicin, which is in line with our results [18,51,52,53,54,55,56,57]. Some studies reported that *A. butzleri* is susceptible to ciprofloxacin [18,31,52,58], whereas other studies reported resistance [8,51,54,55]. Contradictory to our results, previous studies described *A. butzleri* isolates as highly resistant to ampicillin [18,51,52,53,54,56,57]. Strain 16CS0821-2 showed resistance to erythromycin, doxycycline, tetracycline, cefotaxime and streptomycin, whereas strain 16CS0817-2 was only resistant to streptomycin and cefotaxime. Most studies have described resistance against erythromycin [18,31,51,54]. Only a few studies reported susceptibility of *A. butzleri* to erythromycin [52,57,58]. In this study, both phenotypes occurred. The same applies to doxycycline and tetracycline. Strain 16CS0817-2 was susceptible to both antibiotics, while strain 16CS0821-2 was resistant. Similar observations were made in previous studies, in which both phenotypes were described [18,52,53,54,56]. The strains tested here were both resistant to streptomycin. This is in line with the results of some studies, although *A. butzleri* was also found to be susceptible to streptomycin by others [18,51,53,56]. Resistance to cefotaxime has been described before [18,54,56]. The resistance against cefotaxime in *A. butzleri* is well known and is the reason why this antimicrobial agent is often used to supplement culture media to inhibit the growth of unwanted bacteria [56].

However, it is difficult to compare our results with those of previous studies, because of the small number of investigated strains in this study and the different methods used for antimicrobial susceptibility testing. Nevertheless, previous studies already noticed that there is still a lack of standardized methods, specific breakpoints and epidemiological cut-off values regarding antimicrobial resistance of *A. butzleri* isolates [8,21,59].

### 3.2. Genome Assembly

Whole-genome sequencing was done using the Illumina MiSeq instrument, generating 468,526–3,042,934 paired-end reads with a mean sequencing depth of 35–301 reads. The assemblies of the genomes consisted of 89 and 52 contigs with a GC content of 26.97% and 27.01%, respectively (Table 2). The total sequence length was 2,121,905 bp for 16CS0821-2 and 2,432,983 bp for 16CS0817-2.

### 3.3. Taxonomic Classification of the Whole-Genome Sequence Data

**Figure 1:**
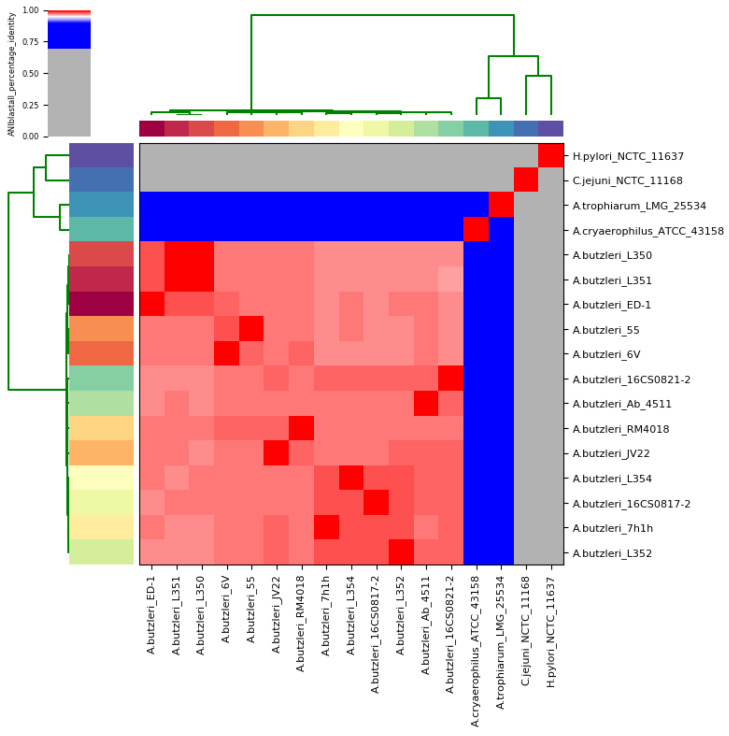
Results of the average nucleotide identity (ANI) analysis. The cells in the heatmap corresponding to an ANI value of 95% and higher are stained red. This indicates that the corresponding strains belong to the same species. Blue colored cells indicate that the corresponding strains do not belong to the same species. The dendrograms (in green; above and on the left side), which were constructed by the simple linkage of the ANIm percentage identities, correspond to the results of the clustering of the ANI values between the used strains [42].

The bioinformatic tool Kraken2 taxonomically classified the two isolates as *A. butzleri*. In order to confirm the taxonomic classification, ANI was calculated between each genome pair of the *A. butzleri* group (Table S1), based on the whole genome sequences. The genomes of *C. jejuni* subsp. *jejuni* NCTC 11168^T^ and *H. pylori* NCTC 11637^T^ were used as outgroups (Table S1). The analysis showed that the two German sequences of *A. butzleri* were very similar (>95%) to the other 11 *A. butzleri* genomes used in this study (Figure 1, Table S2).

In previous studies, it has been shown that if the targeted genomes belong to the same species, the ANI threshold is above 95% [60,61]. The 13 *A. butzleri* sequences had an average pairwise ANI value of 97.6% (range: 96.97 to 99.99%). 16CS0817-2 and 16CS0821-2 shared a mean nucleotide identity of 97.81%. The nearest relatives for 16CS0817-2 were the strains 7h1h and L352 (ANI of 98.28% and 98.25%, respectively), and for 16CS0821-2 the strains L354 and L352 (ANI of 97.96% and 97.89%, respectively). The German isolates had 97.40% and 97.53% nucleotide identity with the reference genome RM4018. The analysis also showed that all *A. butzleri* strains had less than 67% and 64% nucleotide identity with *C. jejuni* subsp. *jejuni* NCTC 11168^T^ and *H. pylori* NCTC 11637^T^, respectively, and less than 78% nucleotide identity with the more closely related species *A. cryaerophilus* ATCC 43158^T^ and *A. trophiarum* LMG 25534^T^ (Table S2). Based on these results, we concluded that the German strains belong to the species *A. butzleri*. In addition, an in silico DDH analysis was performed, which showed DDH values of over 70% when comparing the two German strains and the other 10 *A. butzleri* strains with the reference genome RM4018 (Table 3).

A DDH value of 70% is the recommended standard for delineating distinct species [62,63]. The DDH values of the out-group genomes *C. jejuni* subsp. *jejuni* and *H. pylori* dropped to 21.80% and 25.60%, respectively, when compared to the *A. butzleri* reference genome RM4018, confirming that these strains do not belong to *A. butzleri* (Table 3). Interestingly, the DDH values of *A. cryaerophilus* ATCC 43158^T^ and *A. trophiarum* LMG 25534^T^ were even lower (21.70% and 20.90%, respectively). This result proves that these two strains also do not belong to the species *A. butzleri*. Because of the above mentioned ANI, *A. cryaerophilus* ATCC 43158^T^ and *A. trophiarum* LMG 25534^T^ are more closely related to *A. butzleri* than *C. jejuni* subsp. *jejuni* and *H. pylori*. Therefore, the DDH results supported the result of the ANI analysis, since the recommended DDH cut-off is known to correspond very well to an ANI value of 95% [60,61]. Based on these results, we concluded that our strains belong to the *Aliarcobacter* gen. nov. as *A. butzleri* comb. nov. [2,3,4].

The MLST analysis revealed that the two sequenced strains belong to different sequence types (ST) (Table 4). In each strain, we detected a new allele for the *glyA* locus whose sequences were not identical. Currently, the PubMLST database (accessed on 27.05.2020) contains sequence data of 736 *A. butzleri* isolates from 18 countries with MLST data of only two isolates from Germany and 16 isolates of unknown origin. Of those 736 *A. butzleri* strains, 725 were typed into 680 STs, indicating a high genetic diversity among the *A. butzleri* species. Of note, since January 2019 the PubMLST database for *Aliarcobacter* is no longer curated. Therefore, it was not possible to upload our two sequences and assign them to new STs.

### 3.4. AMR and Heavy Metal Resistance Genes

Although the investigated *A. butzleri* strains showed phenotypic resistance to various antibiotics, no antimicrobial resistance genes were predicted using the public AMR databases. This is probably due to the lack of known antimicrobial resistance-associated genes in *Aliarcobacter* spp. Therefore, we have developed a database specifically for *A. butzleri*, which is based on the current knowledge aboutAMR genes of this species. 

The AMR database created here (ARCO_IBIZ_AMR) contains both specific AMR genes and heavy metal resistance genes, as described before for *A. butzleri* [18,26,27]. In addition, genes were added which, due to their Prokka annotation, were presumed to belong to one of these groups (see Materials and Methods). The final database contains 92 putative AMR genes and 27 potential heavy metal genes.

Of these 119 genes, 77 and 80 genes were detected in 16CS0817-2 and 16CS0821-2, respectively (Table S3). In both tested genomes, 15 efflux pump (EP) systems belonging to different families were identified: a) EP2, EP9, EP12, EP13, EP14, and EP17 (all members of the major facilitator superfamily (MFS)); b) EP8 (belongs to the small multidrug resistance (SMR) family; c) EP3, EP5, EP6 and EP10 (all three belong to the ATP-binding cassette (ABC) family); and d) EP4, EP11, EP15 and EP16 (all members of the resistance modulation division (RND) family) [26]. It is worth mentioning that EP14 and EP17 each consist of only one gene, the *bcr* gene, which is involved in resistance to sulfonamides and bicyclomycin in *Escherichia* (*E*.) *coli* [64]. EP9 confers resistance to fosmidomycin because it contains the *fsr* gene [27]. Of the 19 EP systems described before by Isidro et al., only four (including the Type I secretion system) were not detected [26]. Nevertheless, these findings show that *A. butzleri* harbors all major families of efflux transporters with the exception of the multidrug and toxic efflux (MATE) family [65]. Furthermore, the transcriptional regulator *ohrR* (organic hydroperoxide resistance transcriptional regulator) of the MarR family, which is involved in the response to oxidative stress and the regulation of virulence in *E. coli* [66], and the transcriptional regulator *kstR2* of the TetR/AcrR family, which is associated with cholesterol degradation in *Mycobacterium tuberculosis*, were found but only in the genome of 16CS0821-2 [67]. The survey of the other antibiotic determinants revealed that our two genomes contain additional putative multidrug export ATP-binding/permease proteins (ABU_RS05540, *ybiT1*, *ylmA*, *macB1*) as well as parts of putative EP systems (*acrB*, *tolC*). Strain 16CS0821-2 also carried an outer membrane protein (*oprF3*) and another putative ATP-binding/permease protein (ABU_RS02345) for multidrug export.

Efflux pump mechanisms, ribosomal protection and the enzymatic inactivation of tetracyclines are considered to be the causes of tetracycline resistance [68]. Both the tetracycline efflux pump encoded by the *tetA* gene and the ribosomal protection proteins encoded by, for example, the *tet*(*O*) gene as already described for *Campylobacter* spp., were not present in either genome [69,70]. Therefore, the reason for the resistance of strain 16CS0821-2 to doxycycline and tetracycline must be different. It would be very interesting to investigate this issue in more detail in the future.

Furthermore, we identified five genes, namely *bla2* (putative metallo-hydrolase), *hcpC* (putative β-lactamase), *mrdA*, *pbpB,* and *pbpF* (all penicillin-binding proteins) in both German strains. These genes are suspected to be the reason for the phenotypic resistance to cefotaxime in both isolates. Resistance to β-lactams in Gram-negative bacteria has been described as a combination of the presence and activity of β-lactamase genes and penicillin-binding proteins together with reduced membrane permeability [71]. However, this would contradict the phenotypically determined sensitivity of both strains to ampicillin in this study. An explanation for this result could be the absence of the *bla3* gene, as its presence is associated with ampicillin resistance [26].

Resistance to ciprofloxacin in *Aliarcobacter* spp. is caused by a point mutation (C254T) in the quinolone resistance-determining region (QRDR) of the *gyrA* gene, resulting in an amino acid exchange from threonine to isoleucine (Thr-85-Ile) [29]. In this study, both strains carried neither this characteristic mutation nor any other known mutation (e.g., Asp-89-Tyr [72]). These findings correlate with the phenotype since both isolates were susceptible to ciprofloxacin. However, the *relE* gene, a toxic component of the type II toxin–antitoxin system, was present in both genomes. Overexpression of this gene is associated with the development of persistent resistance to ciprofloxacin and ampicillin [73,74]. Due to the phenotypic susceptibility of both strains to ciprofloxacin and ampicillin, it can be hypothesized that there was no overexpression of the *relE* gene.

Erythromycin resistance could be caused, as previously described for *Campylobacter* spp., by point mutations in the 23S rRNA gene (A2074G, A2074C, A2075G) and/or by amino acid changes in the *rplD* gene (Gly-57-Asp/Val, Gly-67-Val, Ala-71-Asp, Arg-72-Ile, Gly-74-Asp) or *rplV* gene (Gly-74-Ala, Gly-86-Glu, Ala-88-Glu, Ala-105-Met, Thr-109-Ala) [69,70,75,76]. Therefore, we screened our sequences for these modifications. None of the mutations/alterations mentioned above could be detected, not even in the erythromycin-resistant strain 16CS0821-2. The involvement of EP3 was discussed in a previous study because it contains two macrolide export proteins encoded by the *macA1* gene and *macB2* gene [18]. Although the presence of these export proteins could explain the phenotypic resistance of 16CS0821-2 to erythromycin, it contradicts the phenotype of 16CS0817-2. However, the susceptible phenotype of 16CS0817-2 could be explained by the fact that the genes were present, but not expressed. Alternatively, these genes could have a single amino acid substitution that may render them non-functional.

It is noteworthy that the screening for further antibiotic determinants identified additional genes in both isolates that are potentially responsible for resistance to certain antibiotics: *arnB* and *eptA* (resistance to polymyxin), and *rlmN* (resistance to various classes of antibiotics) [18,27]. Two genes, namely *cat3* and *wbpD*, are responsible for resistance to chloramphenicol, but only the *wbpD* gene was detected in strain 16CS0821-2 [26,27]. While three *hipA* genes (*hipA2*, *hipA3* and *hipA4*) were present in strain 16CS0817-2, only the *hipA2* gene could be identified in 16CS0821-2. These *hipA* genes encode a serine/threonine-protein kinase, which is another toxic component of the type II toxin–antitoxin system and is involved in multidrug resistance [77].

The investigation for heavy metal resistance genes revealed a putative copper and arsenic cluster in both strains. This result is in line with previous studies [18,27]. The copper cluster consists of six genes, namely: *copA1* (copper-exporting P-type ATPase A), *copA2* (putative copper-importing P-type ATPase A), *copR* (transcription activator protein), *copZ* (copper chaperone), *csoR* (copper-sensing transcriptional repressor) and cus*S* (sensor kinase). The putative arsenic cluster comprises four genes coding for an arsenic pump membrane protein (*arsB*), an arsenate reductase (*arsC1*), a glutaredoxin arsenate reductase (*arsC2*) and an arsenic resistance protein (ABU_RS02800). Simple arsenic clusters or *ars* operons have been described in a variety of Gram-negative bacteria [78]. 

Furthermore, an almost complete ABC-type transport system for molybdate was detected, which has been described for *E. coli* and *Staphylococcus carnosus* [79,80]. This transport system usually contains three genes: *modA* (molybdate-binding periplasmic protein), *modB* (molybdenum transport system permease protein) and *modC* (cytoplasmic ATPase). Although no *modC* gene was identified in this study, we were able to detect the *mopA* gene (regulator of *modABC*), which was previously described in *Rhodobacter capsulatus* [81]. 

In addition, we detected a mechanism for the export of cadmium, zinc and cobalt in both genomes, which included: a cadmium, zinc and cobalt transporting ATPase (*cadA*) and a cadmium, cobalt and zinc/H^+^-K^+^ antiporter (*czcD*). These results are concordant with previous studies [18,27]. The cobalt–zinc–cadmium resistance proteins encoded by the *czcA* and *czcB* genes were not present in either genome. However, three genes (*czcR1*, *czcR2*, *czcR3*) for the transcription activator protein CzcR were identified. In both strains, the genes of four additional transporters were present: a mercuric transporter (*merT*), a zinc transporter (*zntB*, *ctpC*) and a magnesium and cobalt efflux protein (*corC*) [27].

### 3.5. Virulence-Associated Genes

In this study, a second database was compiled: ARCO_IBIZ_VIRULENCE. This database contains 148 potential virulence determinants including flagellar genes (*n* = 36), chemotaxis system genes (*n* = 8), urease cluster genes (*n* = 6), putative capsule cluster genes (*n* = 7), type IV secretion system (T4SS) genes (*n* = 55), lipid A cluster genes (*n* = 12) and other virulence genes relevant for adherence, invasion and iron absorption (*n* = 24). Most of those virulence genes have been described before for *A. butzleri* [18,26,27]. Of these 148 putative virulence genes, 85 and 78 were present in 16CS0817-2 and 16CS0821-2, respectively (Table S4).

The detection of flagellar genes was expected because *A. butzleri* is a motile bacterium due to the polar flagellum. While in strain 16CS0817-2, all 36 flagellar genes were present, strain 16CS0821-2 only carried 35 genes as the *flaA* gene was not detected. Since the *flaA* gene encodes the same component, the flagellin, as the *flaB* gene, it can be hypothesized that it is not essential for the function of the flagellum. These results are similar to those obtained in previous studies [18,26].

Both genomes presented a complete chemotaxis and urease cluster. The existence of both clusters is consistent with the results of previous studies [26,31]. Although the chemotaxis system was present, only one of the two chemotaxis-associated genes (*luxS*, *ccp* (formerly *docA*)) was detected in both strains—the *luxS* gene. The detection of the urease cluster supports the result of the phenotypic urease assay in a previous study [26]. Therefore, it could be hypothesized that *A. butzleri*, in contrast to *A. cryaerophilus*, can metabolize urea and survive in an environment with a low pH level, similar to *H. pylori* [82].

We highlight the detection of a putative lipid A cluster in both strains. This cluster contains genes encoding the enzymes for the biosynthesis of lipid A as well as those for the regulatory proteins. Together with an oligosaccharide core and the O-antigen polysaccharide, lipid A forms the lipopolysaccharide (LPS), an endotoxin, in Gram-negative bacteria [83]. Lipid A is the only region of the LPS that is recognized by the innate immune system and is capable of triggering a strong immune response in humans and animals [84]. The putative lipid A cluster contains eight genes that are responsible for lipid A biosynthesis: *lpxA*, *lpxB*, *lpxC*, *lpxD*, *lpxH*, *lpxK*, *lpxP*, and *waaA*. *LpxP* is a paralogue of *lpxL* and encodes the palmitoleolytransferase, which is induced by low temperatures. In *A. butzleri*, the presence of *lpxP* could be responsible for the adaptation of growth to low temperatures, thus enabling survival outside a host, as described for *E. coli* [85]. In a previous study, it was reported that several Gram-negative bacteria can modify their lipid A structure. Modifications can lead to higher resistance to cationic antimicrobial peptides of the host and to lower receptor recognition. The regulation of such modifications is mediated by a two-component system called PhoP–PhoQ [83]. Only one of the *phoP* genes, *phoP3* (encoding the virulence transcriptional regulatory protein), was present in our strains. This finding, as well as the absence of the *phoQ* gene, suggests that our strains were not able to change their lipid A structure or use a hitherto unknown mechanism.

The two heptosyltransferases I and II encoded by *rfaC* (formerly *waaC*) and *rfaF* (formerly *waaF*) were detected in both investigated strains. These heptosyltransferases are involved in the biosynthesis of the LPS core, as described in previous publications [27,86]. Although heptosyltransferases are not essential, their absence leads to structural changes in the outer membrane (reduced protein content; increased sensitivity towards hydrophobic agents) and sometimes to a rough phenotype [87].

The putative capsule cluster, as described before, was not present in our genomes [26]. A complete T4SS was also not found in both strains as only a few genes were present: eight in 16CS0817-2 and three in 16CS0821-2.

The following virulence genes associated with cell adhesion and cell invasion were present in both genomes: *ciaB* (host cell invasion protein), *oprF2* (formerly *cadF*; fibronectin-binding protein), *cj1349* (fibronectin-binding protein), *degP* (formerly *htrA*; chaperone involved in adhesion folding), *tlyA* (hemolysin), *pldA* (outer membrane phospholipase A), *iamA* (invasion-associated gene) and *murJ* (formerly *mviN*; integral membrane protein of murein biosynthesis). The presence of virulence-associated genes was first described in connection with the presentation of the complete genome sequence of *A. butzleri* RM4018 [31]. In this study, homologs of the virulence genes *cadF*, *ciaB*, *cj1349*, *mviN*, *pldA,* and *tlyA* of *C. jejuni* were detected in *A. butzleri*. In addition, four further virulence-associated genes (*irgA*, *iroE*, *hecA*, *hecB*) were discovered [31]. In subsequent studies, the same six virulence-associated genes were repeatedly detected in almost all *A. butzleri* strains [30,56,88,89]. Only Fanelli et al. found *iroE* in two isolates together with *ciaB*, *oprF2*, *cj1349*, *murJ*, *pldA,* and *tlyA* [18]. Our results are therefore more or less in agreement with those of previous studies. According to already existing studies, the virulence determinants *irgA* (now *cirA1*) and *iroE* (now *besA*), which are associated with uropathogenicity in *E. coli*, are detected less frequently than the six virulence genes mentioned above [26,31]. This is consistent with our results since only the *besA* gene was present in both strains. Interestingly, another *cirA* gene, the *cirA2* gene, was detected in both strains. This gene had the same Prokka annotation as *cirA1*—colicin I receptor, indicating that these two genes may be involved in iron uptake [90]. Surprisingly, a third *cirA* gene, *cirA3* (formerly *cfrB*), was identified in 16CS0817-2. The former *cfrB* gene is also involved in iron absorption [26]. The ferric uptake is regulated by the *fur* gene, which was present in both genomes [26,90]. In this study, neither the *hecA* (now *cdiA*; filamentous hemagglutinin) nor the *hecB* (now *shlB*; hemolysin transporter) were detected. These genes are rare, although previous studies do not provide consistent data on the presence of *hecB* [18,30,56,88,89].

Finally, we highlight the detection of a) the conserved virulence factor B (*cvfB*), which is known to regulate the expression of virulence factors in *Staphylococcus aureus* [91], b) the virulence protein (*voc*), and c) the virulence regulator transcription activator (*virF*), which has been associated with the regulation of plasmid-transmitted virulence genes in *Shigella flexneri* [92,93], in both analyzed genomes. In contrast, the virulence sensor protein encoded by the *bvgS* gene was not present [27].

## 4. Conclusions

In this study, two *A. butzleri* isolates from a water poultry farm in Germany were sequenced and the taxonomical analyses of their whole-genome sequences revealed that both strains belong to *Aliarcobacter* gen. nov. as *A. butzleri* comb. nov.

To the best of our knowledge, this study presents the first AMR and virulence database for *A. butzleri* only. These databases enable researchers to predict genetic virulence and antimicrobial resistance faster in the future. Nevertheless, the phenotypic determination of antimicrobial resistance should continue to be carried out, as the genotype only corresponds to the phenotype to a limited extent.

Additionally, this is, to the best of our knowledge, the first report about the identification of a lipid A cluster in *A. butzleri*.

## Figures and Tables

**Table 1:** Antimicrobial resistance profiles of *A. butzleri* strains 16CS0817-2 and 16CS0821-2.

Strain	ERY	CIP	DC	TET	GM	SM	AMP	CTX
16CS0817-2	S (2 mg/L)	S (0.03 mg/L)	S (1.5 mg/L)	S (2 mg/L)	S (1.5 mg/L)	R (6 mg/L)	S (4 mg/L)	R (32 mg/L)
16CS0821-2	R (40 mg/L)	S (0.38 mg/L)	R (4 mg/L)	R (3 mg/L)	S (1.5 mg/L)	R (12 mg/L)	S (4 mg/L)	R (32 mg/L)

S = sensitive; R = resistant; minimal inhibitory concentration in brackets (ERY = erythromycin, CIP = ciprofloxacin, DC = doxycycline, TET = tetracycline, GM = gentamicin, SM = streptomycin, AMP = ampicillin, CTX = cefotaxime).

**Table 2:** Summary of the assembly results of the *A. butzleri* strains 16CS0817-2 and 16CS0821-2 using SPAdes (v. 3.12.0), Quast (v. 4.3) and Prokka (v. 1.14).

Assembly	16CS0817-2	16CS0821-2
Total length	2,432,983	2,121,905
GC (%)	26.97	27.01
Nr. of contigs	89	52
Largest contig	159,485	177,585
N50	62,983	69,691
Predicted genes	2500	2159
CDS	2451	2110
rRNA	3 (5S,16S, 23S)	3 (5S, 16S, 23S)
tRNA	46	46
tmRNA	1	1

**Table 3:** Results of the in silico DNA–DNA hybridization (DDH).

Strain	Species	*Aliarcobacter butzleri* RM4018		
		Identities/HSP Length		Difference in % G + C (≤1 Either Distinct or Same Species; >1 Distinct Species)
		Distance	DDH Estimate (GLM-Based)	
16CS0817-2	*A. butzleri*	0.0248	78.80% [75.8–81.4%]	0.07
16CS0821-2	*A. butzleri*	0.0238	79.70% [76.7–82.3%]	0.04
6V	*A. butzleri*	0.0222	81.00% [78–83.6%]	0.20
55	*A. butzleri*	0.0219	81.20% [78.3–83.8%]	0.26
7h1h	*A. butzleri*	0.0242	79.30% [76.3–81.9%]	0.01
Ab_4511	*A. butzleri*	0.0239	79.50% [76.6–82.2%]	0.09
ED-1	*A. butzleri*	0.0247	78.90% [75.9–81.6%]	0.16
JV22	*A. butzleri*	0.0218	81.40% [78.5–83.9%]	0.03
L350	*A. butzleri*	0.0254	78.30% [75.3–81%]	0.10
L351	*A. butzleri*	0.0253	78.40% [75.4–81.1%]	0.06
L352	*A. butzleri*	0.0237	79.70% [76.8–82.4%]	0.03
L354	*A. butzleri*	0.0252	78.50% [75.5–81.2%]	0.11
ATCC 43158	*A. cryaerophilus*	0.2025	21.70% [19.4–24.1%]	0.42
LMG 25534	*A. trophiarum*	0.2101	20.90% [18.7–23.3%]	1.17
NCTC 11168	*C. jejuni* subsp. *jejuni*	0.2010	21.80% [19.6–24.3%]	3.50
NCTC 11637	*H. pylori*	0.1699	25.60% [23.2–28.1%]	11.78

HSP = high-scoring segment pair; GLM = generalized linear model; ATCC = American Type Culture Collection; LMG = Bacterial Collection of the Laboratory of Microbiology of the University of Ghent; NCTC = National Collection of Type Cultures; A. = *Aliarcobacter*; C. = *Campylobacter*; H. = *Helicobacter.*

**Table 4:** Results of the multilocus sequence typing (MLST). Allelic profiles of the *A. butzleri* isolates 16CS0817-2 and 16CS0821-2.

Strain	*aspA*	*atpA*	*glnA*	*gltA*	*glyA*	*pgm*	*tkt*	ST
16CS0817-2	177	39	40	123	new allele	194	24	new
16CS0821-2	47	217	4	129	new allele	123	37	new

## Supplementary Materials

The following are available online at https://www.mdpi.com/2073-4425/11/9/1104/s1, Table S1: Genomes used in this study, Table S2: Results of the ANI analysis, Table S3: Presence and absence of AMR genes in the *A. butzleri* strains 16CS0817-2 and 16CS0821-2, Table S4: Presence and absence of virulence genes in the *A. butzleri* strains 16CS0817-2 and 16CS0821-2.
